# Improving the reporting quality of intervention trials addressing the inter-individual variability in response to the consumption of plant bioactives: quality index and recommendations

**DOI:** 10.1007/s00394-019-02069-3

**Published:** 2019-09-06

**Authors:** Marina Nikolic, Aleksandra Konic Ristic, Antonio González-Sarrías, Geoffrey Istas, Mireia Urpi-Sarda, Margherita Dall’Asta, Laurent-Emmanuel Monfoulet, Lieselotte Cloetens, Banu Bayram, Maria Rosaria Tumolo, Mihail Chervenkov, Egeria Scoditti, Marika Massaro, Noemi Tejera, Desislava Abadjieva, Karen Chambers, Irena Krga, Francisco A. Tomás-Barberán, Christine Morand, Rodrigo Feliciano, Rocío García-Villalba, Mar Garcia-Aloy, Pedro Mena

**Affiliations:** 1 Institute for Medical Research, University of Belgrade, Belgrade, Serbia; 2 UCD Institute of Food and Health, University College Dublin, Belfield, Dublin, Ireland; 3 Laboratory of Food and Health, Research Group on Quality, Safety and Bioactivity of Plant Foods, CEBAS-CSIC, Murcia, Spain; 4 Department of Nutritional Sciences, Faculty of Life Sciences and Medicine, School of Life Course Sciences, King’s College London, London, UK; 5 Biomarkers and Nutrimetabolomic Laboratory, Department of Nutrition, Food Sciences and Gastronomy, XaRTA, INSA, Faculty of Pharmacy and Food Sciences, University of Barcelona, Santa Coloma De Gramenet, Spain; 6 CIBER de Fragilidad y Envejecimiento Saludable (CIBERFES), Instituto de Salud Carlos III, Barcelona, Spain; 7 Human Nutrition Unit, Department of Food and Drugs, University of Parma, Medical School Building C, Via Volturno, 39, 43125 Parma, Italy; 8 Unité de Nutrition Humaine (UNH), Institut National de la Recherche Agronomique (INRA), Université Clermont Auvergne, CRNH Auvergne, Clermont-Ferrand, France; 9 Biomedical Nutrition, Pure and Applied Biochemistry, Lund University, Lund, Sweden; 10 Department of Nutrition and Dietetics, University of Health Sciences, Istanbul, Turkey; 11 Research Unit of Brindisi, Institute for Research on Population and Social Policies, National Research Council, Brindisi, Italy; 12 Faculty of Veterinary Medicine, University of Forestry, Sofia, Bulgaria; 13 Institute of Neurobiology, Bulgarian Academy of Sciences, Sofia, Bulgaria; 14 Institute of Clinical Physiology (IFC), National Research Council (CNR), Lecce, Italy; 15 Department of Nutrition and Preventive Medicine, Norwich Medical School, University of East Anglia, Norwich, UK; 16 Institute of Biology and Immunology of Reproduction, Bulgarian Academy of Sciences, Sofia, Bulgaria; 17 Quadram Institute Bioscience, Norwich Research Park, Norwich, UK; 18 Division of Cardiology, Pulmonology, and Vascular Medicine, Medical Faculty, University of Duesseldorf, Dusseldorf, Germany

**Keywords:** Clinical trials, Reporting quality, Inter-individual variation, Quality index, Plant bioactive, Recommendations, Guidelines

## Abstract

**Purpose:**

The quality of the study design and data reporting in human trials dealing with the inter-individual variability in response to the consumption of plant bioactives is, in general, low. There is a lack of recommendations supporting the scientific community on this topic. This study aimed at developing a quality index to assist the assessment of the reporting quality of intervention trials addressing the inter-individual variability in response to plant bioactive consumption. Recommendations for better designing and reporting studies were discussed.

**Methods:**

The selection of the parameters used for the development of the quality index was carried out in agreement with the scientific community through a survey. Parameters were defined, grouped into categories, and scored for different quality levels. The applicability of the scoring system was tested in terms of consistency and effort, and its validity was assessed by comparison with a simultaneous evaluation by experts’ criteria.

**Results:**

The “POSITIVe quality index” included 11 reporting criteria grouped into four categories (Statistics, Reporting, Data presentation, and Individual data availability). It was supported by detailed definitions and guidance for their scoring. The quality index score was tested, and the index demonstrated to be valid, reliable, and responsive.

**Conclusions:**

The evaluation of the reporting quality of studies addressing inter-individual variability in response to plant bioactives highlighted the aspects requiring major improvements. Specific tools and recommendations favoring a complete and transparent reporting on inter-individual variability have been provided to support the scientific community on this field.

**Electronic supplementary material:**

The online version of this article (10.1007/s00394-019-02069-3) contains supplementary material, which is available to authorized users.

## Key messages

- The reporting quality of human studies on inter-individual variation in response to plant bioactives is generally low and should be significantly improved.
- There is no specific guidance for reporting studies on inter-individual variation in response to plant bioactives.
- The assessment of reporting quality using a scale approach is considered a valuable tool in assessing compliance with the recommendations in the submission phase or during the reviewing process. It also provides a quantitative measure of the quality of studies to be used in meta-analysis.
- Eleven reporting criteria were developed and supported by detailed definitions and guidance for their scoring.
- The POSITIVe quality index was tested and demonstrated to be valid, reliable, and responsive.
- The use of the quality index and its supporting explanatory material (dictionary) as a guide for researchers, peer-reviewers, and journal editors will foster further complete and transparent reporting of data on inter-individual variability.
- The criteria used in the quality index can serve as additional guidance to inform the design and conduction of further studies on inter-individual variations in response to plant bioactives.
- Better reporting is expected to lead to a better understanding of the mechanisms and factors involved and thus better study designs with greater impact on policies and practice.

**Electronic supplementary material:**

The online version of this article (10.1007/s00394-019-02069-3) contains supplementary material, which is available to authorized users.

## Introduction

A large body of evidence supports the notion that bioactive compounds present in plant foods [e.g., (poly)phenols, carotenoids, glucosinolates, etc.] have numerous beneficial effects on human health, underlying the association between the habitual consumption of plant-based diets and the reduced risk of age-related chronic diseases [1, 2]. However, data from clinical trials aiming to establish the cause–effect relationship are often inconclusive and even contradictory [3, 4]. It has been suggested that this may be, at least partly, the result of the differences in the bioavailability of plant bioactives among individuals, as well as the variation in their effects on specific functional biomarkers, either physiological or biomarkers of risk [5]. A clear understanding of all the factors responsible for the inter-individual variability (IIV) in response to plant bioactives is the vital part of knowledge that is still lacking, yet it is considered crucial for the ultimate positioning of plant bioactives on the roadmap to optimal health. To gain this seminal knowledge, the complex interactions of plant bioactives with the factors that drive the genotype and phenotype of individuals should be assessed [5–7].

A recent series of systematic reviews and meta-analyses of human studies addressing the IIV in response to different plant bioactives concluded that the number of trials reporting between-subject variations is, in general, very low [8–10]. At the same time, since most studies are not initially designed to address IIV, they are often underpowered within groups and unbalanced between them, with multiple sources of bias including selective reporting, insufficient description of subjects’ characteristics and inaccurate reporting on statistics, as well as providing inadequate conclusions on the observed effects. Consequently, although in most of the cases the quality of protocol design and reporting of the initial study respond sufficiently to general recommendations and requirements [11–13] studies were often considered as non-adequate in terms of post hoc subgroup statistics, regressions, or other similar approaches in the analysis. It is thus evident that the design and reporting of human trials addressing the between-subject variation are crucial and should be improved. Moreover, a better way of designing and reporting human intervention trials on the effect of plant bioactives while considering individual differences would help to further summarize and analyze aggregated data through systematic reviews and meta-analyses. An inadequate reporting quality of studies often makes them non-eligible to be included in meta-analyses or it may introduce a significant bias in the analysis, compromising the accuracy and reliability of the conclusion [10, 14]. Nevertheless, despite the multiple benefits related to appropriate design and reporting of studies dealing with IIV in response to plant bioactive consumption, there is a lack of suitable recommendations and/or guidelines supporting the scientific community and favoring improvements in the way data are assessed and disseminated. Unfortunately, the quality assessment tools that are available to date [11, 15–17] do not fit sufficiently the scientific questions and purposes of this specific type of reporting of intervention trials on food products or supplements rich in plant bioactives.

This study aimed at proposing a specific quality index (QI) as a tool to be used in the assessment of the reporting quality of human intervention trials addressing the IIV in response to plant bioactive consumption (so-called the “POSITIVe quality index”). The IIV QI was tested for its reliability, validity and responsiveness. The steps carried out to develop this QI have also been thoroughly reported as a roadmap and recommendations for its application to better design and report human intervention trials in the field have been provided.

## Methods

### Rationalizing the need to develop an additional tool to assess reporting quality and expert agreement

This study was performed as part of the COST Action FA1403 POSITIVe (https://www6.inra.fr/cost-positive), a collaborative and multidisciplinary pan-European network of more than 300 researchers. One of the main objectives of the Action was to identify the main factors responsible for the observed IIV in response to plant food bioactives intake, based on available scientific evidence and generated new knowledge. While dealing with this objective in the early phases of the Action, it was noted that the reporting quality of human studies in the field was often limited and inadequate. This issue was further discussed by ten experts of the Action Think Tank Group (addressed in the following text as “score developers”). One of the main reasons hypothesized was the lack of generally accepted and routinely applied recommendations to report between-subject variation, either as primary or post hoc analysis. The strategy to solve this gap was defined and it consisted of several initial steps: (1) to review the existing literature and identify previous relevant guidance and assessment tools; (2) to seek relevant evidence on the quality of reporting in published research articles; (3) to identify key information related to the potential sources of bias in such studies; and (4) to use the POSITIVe Action network for expert opinions and resources.

The literature search was performed through the Equator Network Library resources (http://www.equator-network.org/library/), Medline, Embase and Cochrane databases, and through a Pubmed search using specified search terms (e.g., “quality assessment”, “tools”, “reporting”, “guideline(s)”, “inter-individual”, “quality index”, “recommendation”).

The retrieved and critically selected literature included: (1) tools often used for assessing reporting quality of individual studies [18] or tools for assessing reporting bias during systematic reviews and meta-analyses [19]; (2) a scarce number of available resources that specifically address IIV in clinical trials [20–23]; (3) papers retrieved during the ongoing systematic searches and meta-analyses on IIV in response to plant bioactives; (4) two position papers of the Action [5, 6]; (5) several papers on “controversies” on clinical trial methodology and reporting [24–26]; and (6) available strategies on creating and developing tools and guidelines [27–29].

**Fig. 1:**
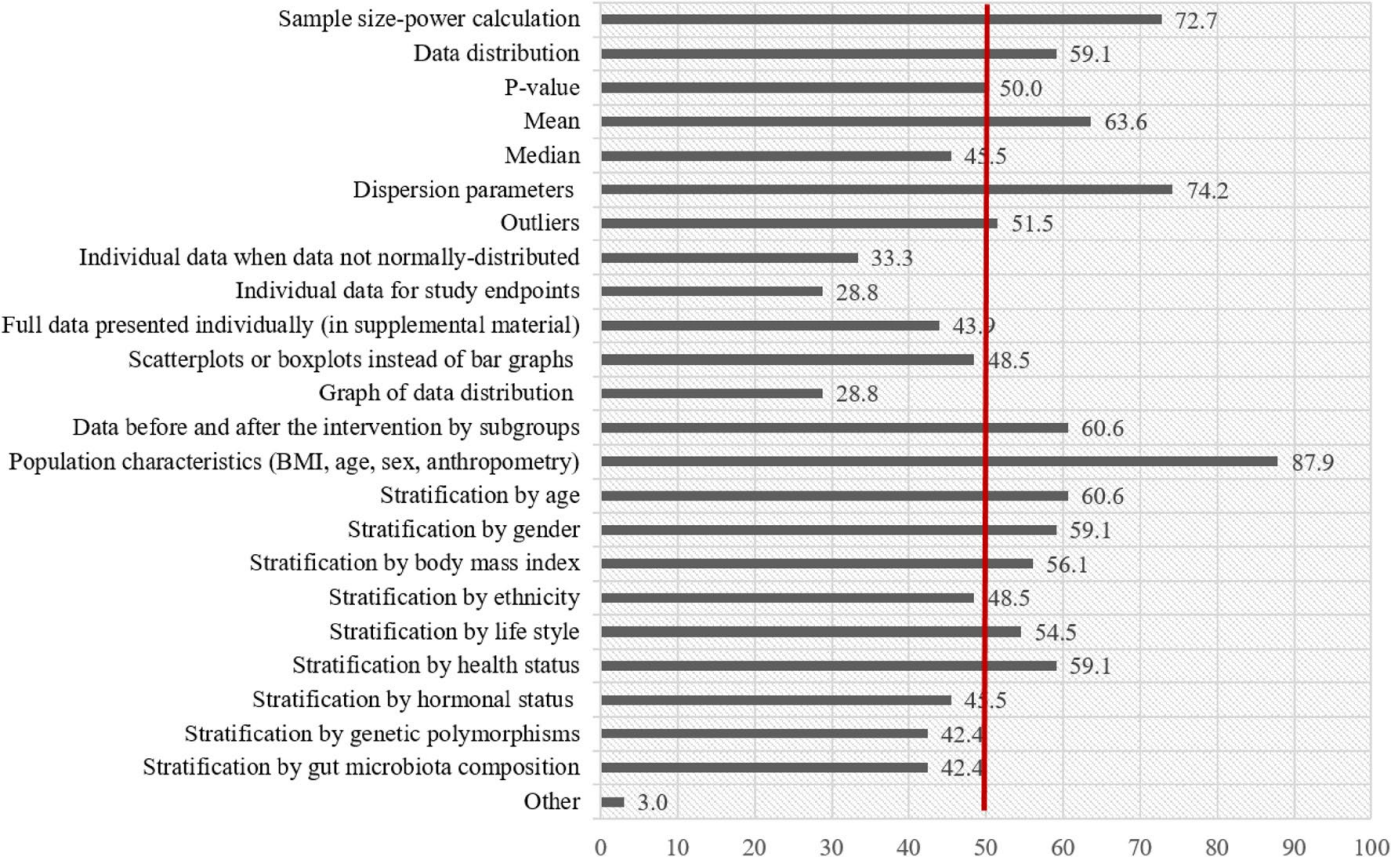
Percentage of experts who considered the listed parameters important to be reported when assessing inter-individual variability in response to consumption of plant food bioactives

Based on the information from the extracted literature, the “score developers” made a list with up to 23 criteria addressing all relevant parameters that may be considered as specific for data reporting in studies assessing IIV in response to plant bioactives intake (Fig. 1).

In the next step, the “score developers” designed a questionnaire entitled “How to Report Inter-Individual Variability in Publications” that was sent to 311 members of the POSITIVe network. It included questions addressing expert opinion on (i) the need for a specific assessment tool to be developed; (ii) the familiarity with the Jadad score used to assess the quality of reports of randomized trials; (iii) the need for extension for this specific purpose; (iv) the interest of reviewers and journal editors in the network to adopt and implement a quality index score (QIS) in the reviewing process; and, as a crucial part, (v) members were asked to select a number of criteria they consider relevant enough to be included in the score, from the list of 23 criteria made by the “score developers”.

### Selection of categories and parameters to be addressed in the assessment and development of the IIV quality index scoring system

After collection and evaluation of the questionnaires, “score developers” defined the categories and selected the parameters within each category to be included in the score. Parameters selected by 50% or more experts were directly taken into consideration to be included in the IIV QI. Parameters that were considered important by 40–50% of experts were additionally discussed and evaluated by score developers, while parameters selected by less than 40% of participants were excluded from the score. As a result, a list of 11 parameters grouped in 4 categories was defined for the design of the QI and the development of recommendations/guidelines for data reporting on IIV (Table 1).

**Table 1:** Dictionary of conditions related to data reporting on IIV and associated scores for the evaluation of quality of data reporting in intervention studies dealing with plant bioactives

Category	Parameters	Condition	Score
Statistics	Sample size-power calculation	Authors reported on: (1) power calculation focused on assessing inter-individual variation based on primary OR the most limiting outcome AND (2) on all the data used in power calculation (% of statistical power, significance level, expected dropout rate, expected difference between groups of the mean or % with the event) AND (3) the resulting sample size per each group	1
		Authors did not describe sample size taking into account all the three previous conditions	0
	Data distribution	Authors specified the test used for normality (OR indicate something related to data normality, for instance, log transformation)	1
		Authors did not report any information related to the normality or distribution of the data in general	0
	*p* value	Authors reported *p* value that support IIV (e.g., *p* value related to the examination of differences between two or more factors affecting IIV such as sex, age, genotypes, etc.)	1
		Authors did not report any *p* values related to the IIV	0
	Effect size	Authors reported the magnitude of the IIV for the selected outcome(s) by standardized mean differences as an index of effect size (Cohen’s d, % of coefficient of variation, etc.) or any parameters related to the effect size suitable for the conducted statistical tests	1
		Authors did not indicate any parameters related to the effect size i.e., magnitude of the IIV for the selected outcome(s) (any of standardized mean differences was not reported)	0
Reporting	General characteristics of the subgroups where IIV was evaluated	Authors reported on one or more general characteristics (for instance, ethnicity, BMI, age, gender, smoking status, etc.) for each of the subgroups where IIV was evaluated	1
		Authors did not report any of general characteristics for the study sample (for instance, ethnicity, BMI, age, gender, smoking status, etc.) on each subgroup where IIV was evaluated	0
	Data reporting for end-points by subgroups	Both pre- and post-intervention data (or post-intervention data as % change with respect to a provided baseline value) were reported for different subgroups where IIV was evaluated	1
		Post-intervention data (or % change without a provided baseline value) are provided by each subgroup where IIV was evaluated	0.5
		Neither pre- nor post-intervention data were reported for different subgroups where IIV was evaluated	0
	Measures of central tendencies and dispersion parameters	Authors reported on one or more measures of central tendencies (mean, median, etc.) AND one or more dispersion parameters (standard deviations, standard error, interquartile range, 95% confidence interval, etc.) for EACH subgroup where IIV is evaluated	1
		Authors did not report any measures of central tendencies or dispersion measures for subgroups where IIV was evaluated, regardless of reporting these parameters for the total sample	0
	Outliers	Authors indicated outliers AND described them (explained the reason for treating them as outliers)	1
		Authors indicated that outliers existed and that were excluded from the analysis but without describing them	0.5
		Authors did not indicate any information related to outliers	0
Data presentation	Tables	Tables contain additional measures of variability (min–max, interquartile range, outliers values, etc.) or individual measures (responders/non-responders, etc.)	1
		Tables did not contain any extra measures of variability (min–max, interquartile range, outliers values, etc.) nor individual measures (responders/non-responders, etc.)	0
	Graphs	Authors presented data for the primary outcome by scatterplots, boxplots or heat maps	1
		Authors presented data by histograms for a primary outcome OR as scatterplots and boxplots for secondary outcomes	0.5
		Data are graphically presented as bar chart, curves, etc. (for any of the study point-before or after the intervention) but not as scatterplots, boxplots, heat maps or histograms	0
Individual data availability	Presentation of full data and population characteristics	Authors provided individual data for each end-point, together with the characteristics of the samples on the individual level, in the paper or in the supplemental material	2
		Authors provided individual data at each end-point (even presented in the figures) but without any additional characteristics of the sample on the individual level	1
		Authors did not provide individual data	0

The next step was the creation of the first version of a dictionary, with detailed definitions of the conditions related to data reporting for each parameter and with assigned marks, as a base for the calculation of the QIS. Marks were assigned to each parameter and its related conditions while reporting as follows: if a particular parameter is not considered in the study at all, its score is 0; if it is reported but not informative enough to completely describe IIV, its score is 0.5; and, if it is considered and completely illustrate IIV, its score is 1. The exception to this scale was the last category, related to the individual data availability. Considering the importance of this parameter for the accurate assessment of IIV, developers modified the scale to 0–1–2, to increase the weight of this parameter in the score. The dictionary of the QI and parameter marks are presented and fully described in the results section.

### Testing the IIV quality index score validity, reliability, and responsiveness

In the next phase, the QI was tested and validated by evaluating and scoring existing studies, on the basis of their comprehensiveness of data reporting on IIV. The evaluation and scoring were performed by nineteen experts (“evaluators”), all members of the Think Tank Group of the Cost Action POSITIVe. Evaluators had previous experience in clinical trials on plant bioactives, as shown by their publication records, and they were all involved in the ongoing systematic reviews and meta-analyses performed as part of the objectives of the Action [5, 8–10]. The evaluators screened the dossiers of peer-reviewed articles retrieved within the systematic searches they were involved in and identified 30 articles that specifically reported IIV in response to plant bioactives consumption. The total number of 30 studies was considered sufficient for the purpose of testing the IIV QI.

In the first step, the comprehensiveness of the parameters and the dictionary was tested in a pilot trial by evaluating five studies. These studies were selected from the comprehensive list of 30 studies, using computer-assisted random selection from 2 subgroups, resulting in 2 random studies on bioavailability and 3 on the effects of plant bioactives. Each study was evaluated independently by two or three different evaluators, and the final scoring was done based on their consensus. After the pilot testing phase, all evaluators provided their critical opinion to the developers and helped in revising the dictionary to be clear and user friendly. Revisions were related to the definitions of conditions and regrouping parameters between categories. Once the final version of the dictionary for the QI was created, 30 studies, (including 5 using in the test phase) were evaluated and scored in the same way as in the testing phase [30–59].

After the evaluation, data reporting quality of each study was assessed by the total score and four sub-scores related to the four categories considered: Statistics, Reporting, Data presentation and Individual data availability (Table 1). In addition to the evaluation carried out by employing the IIV QIS, evaluators assessed the overall quality of data reporting on IIV for each study using a qualitative scale and characterizing them as weak, mild, or good based on their personal opinions as experts. QI was then validated by comparing these two methods of evaluation.

### Statistics

Accuracy of QI was examined by analyzing relations between the quality of the studies as assessed by experts using quantitative scale (weak, mild, and good) and the QIS. The ordinal variable was created for the quality level assessed by experts (weak = 1, mild = 2, good = 3). The overall quality score was calculated for each study as the sum of all marks given to each study divided by 11 (number of selected parameters). Completeness of reporting within each defined category was calculated for each study as the sum of all marks assigned to the parameters from the category divided by the number of parameters for that particular category. In this way, completeness of reporting was standardized for all categories. Spearman’s correlation coefficient was calculated for the relation between overall IIV QIS and the quality level assessed by experts. Cohen’s kappa coefficient was calculated to test the agreement between tertiles of the overall IIV QIS and quality levels assessed by the experts. Impact of completeness of each defined category within the QI on the quality levels assessed by experts was also assessed by the Spearman correlation coefficient. A value of *p *< 0.05 was taken to indicate a significant result. All analyses were performed using SPSS statistical software (IBM SPSS statistics 20, SPSS Inc., Chicago, IL USA).

## Results

### Questionnaire results

The questionnaire ‘How to Report Inter-Individual Variability in Publications’ was answered by 66 experts involved in the POSITIVe network, resulting in a response rate of 21%. The majority of responders (96%) considered the development of the QI important for the assessment of the quality of data reporting on IIV in publications dealing with plant bioactive consumption. In general, experts had different approaches during the selection of parameters from the list. The selection of fewer than ten parameters (more critical approach) was observed in 44% (*n* = 29) of those who answered the questionnaire. This subgroup of experts was focused on statistical parameters (sample size, normality, *p* value related to IIV), parameters related to the measures of central tendencies and dispersion, and parameters related to the population characteristics and stratification by different factors that could affect IIV. The rest of the experts were prone to choose more than 10 parameters, 24% of them selected between 11 and 15 parameters, while 32% of them considered more than 15 parameters as important. Answers provided by all responders were taken into consideration in further developing the QI. Percentage of experts, who considered the listed parameters important to be reported when assessing IIV in response to plant bioactives, is presented in Fig. 1. Sample size calculation, dispersion parameters, and population characteristics were selected by more than 70% of experts, as the most important parameters related to IIV. Data distribution, *p* value, mean, outliers, data before and after intervention by subgroups, and stratification by different factors (age, gender, body mass index, lifestyle, and health status) were considered important by more than 50% of experts. However, stratification by ethnicity, hormonal status, polymorphism, and gut microbiota composition were selected by 40–50% of experts, as well as median, full data presented on individual level provided in the supplementary material, and data depicted in scatter or box plots. Graphical presentation of data distribution, individual presentation of non-normally distributed data, and individual data for study end-point were assessed as important by less than 35% of experts. Additional parameters under the option “other” were suggested by 12 experts. Only one of them (coefficient of variation) was related to data reporting from the statistical point of view, while all other parameters were more general and not directly related to the data reporting but factors important for IIV, like dietary habits, physical activity, etc. The experts were asked about the Jadad scale for reporting randomized trials, and 36.4% of them stated that they were familiar with this scale developed in 1996 by Jadad et al. [15] while 59% of responders said that it would be important to supplement the Jadad scale with the QI related to IIV.

Among the experts who answered the questionnaire, there were 16 (24%) members of editorial boards in peer-reviewed scientific journals, with the SJR ranking for these journals being between 0.22 and 1.53 in 2018, including 7 with the SJR above 1. The majority of experts (80%) claimed that journal editors might be interested in the QI to be used during the evaluation process of manuscripts dealing with IIV in response to plant bioactives.

### Development of the quality index and the dictionary as associated explanatory document

The final version of the dictionary with defined conditions for each parameter and assigned scores is presented in Table 1. Sample size/power calculation, distribution of the data (normality), and *p* value related to the IIV were grouped in one category (*Statistics*). Additionally, after testing the first version of the dictionary for the QIS, evaluators stressed the importance of reporting on the effect size of the applied statistical tests, as an important parameter for the complete understanding of the *p* value significance [60]. Finally, the sum of scores based on the first category (*Statistics*) reflects on the quality of data reporting concerning four parameters: sample size/power calculation, data distribution, *p* value, and effect size. All parameters in this category could be assessed by a dichotomous score of 0 or 1.

Another set of six parameters listed in the questionnaire were regrouped into four and integrated into the second category (*Reporting*) (Table 1). Reporting on general characteristics of the subgroups where IIV is generally evaluated (age, gender, body mass index, smoking, etc.) was the parameter most often selected by experts (87.9%). Since they had diverse opinions about stratification according to different characteristics, it was decided to keep this parameter open for any characteristic collected for the possible study subgroups. Reporting on data for study end-points by subgroup (before and after the intervention) was also included within the *Reporting* category. Furthermore, measures of central tendencies and dispersion parameters reported for each subgroup were merged and included in this category as a single parameter. Reporting on outliers was integrated into the *Reporting* category as the fourth parameter. Data reporting on end-points by subgroups and on outliers could be scored by values 0, 0.5, or 1, depending on the comprehensiveness of the reporting (Table 1). On the other hand, reporting on general characteristics and measures of central tendencies and dispersion parameters could be scored by 0 or 1. In conclusion, overall score related to the *Reporting* category reflects the quality of reporting on four parameters: general characteristics of the subgroups, data reporting for end-points by subgroups, measures of central tendencies and dispersion parameters, and outliers.

The third category considered important by developers, taking into account both tables and graphs (Table 1), was *Data presentation*. Though the complexity of tables was not listed as a parameter in the questionnaire, after testing, evaluators suggested to include this parameter. Its purpose is to assess reporting on additional values that could reflect IIV (min, max, interquartile range, number of responders/producers, etc.), apart from common measures of central tendencies and dispersion parameters. Presenting data as scatter plots or box plots instead of bar charts was considered important by 49% of experts. Developers decided to extend this parameter to assess the global quality of graphical data presentation, making a distinction between presenting data on primary and secondary outcomes. It means that, in the final version of the dictionary, the scatter plots, box plots, or heat maps that depict data related to the primary outcome are considered as the most useful (score = 1); histograms depicting primary outcome data or scatter plots/box plots/heat maps depicting secondary outcomes are considered as not so informative but still useful ways of illustrating IIV (score = 0.5); and bar charts, curves, etc. for any outcome are considered as not helpful in the assessment of IIV (score = 0).

The fourth category was related to the availability of individual data, i.e., transparency of analyzed data set and the possibility of further analysis. Developers distinguished three different levels within this category. Individual data available for each end-point reported together with the characteristics of the study participants on an individual level are the most appreciated option (score = 2). Individual data reported for each end-point but without any additional characteristics of study participants are still considered useful but less than the previous option (score = 1), while studies that did not show any individual data are not scored for this category (i.e., score = 0).

### Validation of the quality index-evaluation of collected studies

QIS and categories sub-scores were calculated for 30 studies (Supplementary Table 1). Quality of data reporting on IIV for these studies was additionally evaluated by experts using qualitative scale, and 2 studies were assessed as weak, 12 studies as mild, and 16 studies as good. A weak agreement was found between terciles of overall QIS and the three levels of quality (weak, mild, and good) assessed by experts (Cohen’s *k* = 0.216, *p *= 0.054). The significant agreement between both methods was confirmed by Spearman’s rank correlation coefficient (*r* = 0.697, *p *< 0.001).

**Fig. 2:**
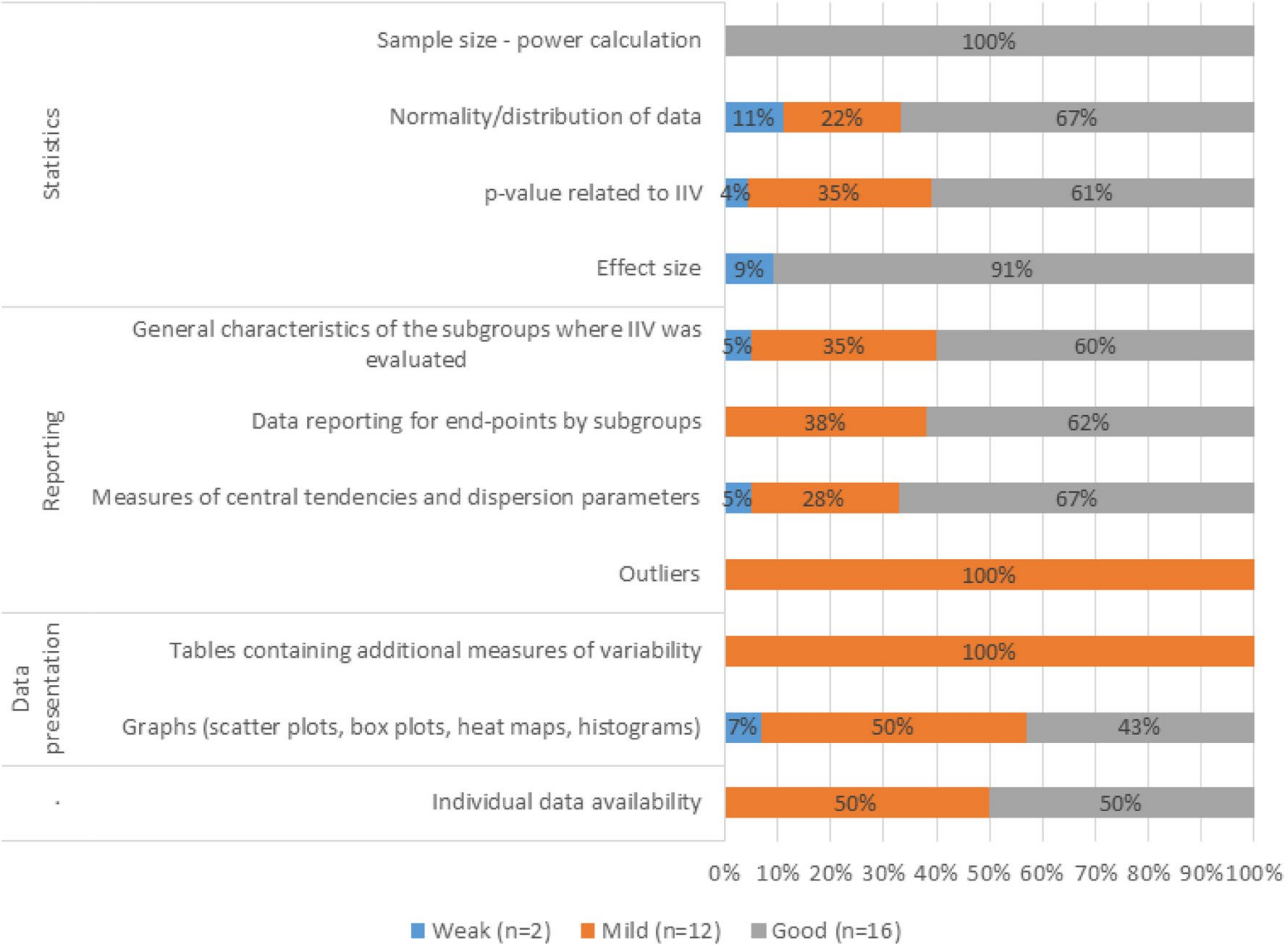
Number and percentage of good, mild, and weak studies with respect to the reported parameters within defined categories

Numbers and percentages of studies that reported any data on the selected parameters (scored either 1 or 0.5 according to the dictionary) are presented in Table 2. Significant correlations were found between the completeness of particular score categories (*Statistics* and *Reporting*) and quality levels assessed by experts (Spearman’s *r* = 0.519, *p *= 0.003; *r* = 0.509, *p *= 0.004, respectively). On the contrary, *Data presentation* category was inversely correlated with the expert’s opinions (*r* = − 0.365, *p *= 0.047). Individual data availability, as an independent parameter, were not analyzed in this way since only four studies provided data, but without additional characteristics of the groups where IIV was evaluated. Weak, mild, and good studies that reported on selected parameters are presented in Fig. 2. *Statistics* category was found as the most important for high-quality data reporting on IIV. All studies assessed as good by experts reported on, at least one, parameter from the *Statistics* category, while 75% of them reported on two or more parameters from this category.

**Table 2:** Reporting on parameters within defined categories [*n* (%)] and completeness of reporting (%) within each category by quality of studies assessed by experts’ opinion

Category	Parameter	Weak (*n* = 2)		Mild (*n* = 12)		Good (*n* = 16)		Total (*n* = 30)		Completeness vs. quality levels assessed by experts	
		*n* (%)	Completeness	*n* (%)	Completeness	*n* (%)	Completeness	*n* (%)	Completeness	Spearman’s correlation coeff.	*p* value
Statistics	Sample size-power calculation	0 (0%)	37.5%	0 (0%)	20.8%	1 (6%)	48.4%	1 (3%)	36.7%	0.519	0.003
	Normality/distribution of data	1 (50%)		2 (17%)		6 (38%)		9 (30%)			
	*p* value related to IIV	1 (50%)		8 (67%)		14 (88%)		23 (77%)			
	Effect size	1 (50%)		0 (0%)		10 (63%)		11 (37%)			
Reporting	General characteristics of the subgroups where IIV was evaluated	1 (50%)	25%	7 (58%)	44.8%	12 (75%)	59.4%	20 (67%)	51.3%	0.509	0.004
	Data reporting for end-points by subgroups	0 (0%)		8 (67%)		13 (81%)		21 (70%)			
	Measures of central tendencies and dispersion parameters	1 (50%)		6 (50%)		14 (88%)		21 (70%)			
	Outliers	0 (0%)		2 (17%)		0 (0%)		2 (7%)			
Data presentation	Tables	0 (0%)	25%	2 (17%)	33.3%	0 (0%)	14.1%	2 (7%)	22.5%	− 0.365	0.047
	Graphs	1 (50%)		7 (58%)		6 (38%)		14 (47%)			
Individual data availability		0 (0%)		2 (17%)		2 (13%)		4 (13%)		–	–

Moreover, 91% of those that reported on effect size were assessed as good by expert’s independent opinion. Only one study reported on sample size as expected (i.e., as described in the dictionary). Studies that reported on data distribution and *p* value related to IIV were assessed as good in 67% and 61% of cases, respectively (Fig. 2). Regarding the *Reporting* category, most of the studies assessed as good by experts took into consideration parameters related to this category (Fig. 2). Results showed that 63% out of 21 studies that reported on data for end-points by subgroups were categorized as good by evaluators, and there were no studies characterized as weak. These results were similar for studies reporting on measures of central tendencies and dispersion parameters by subgroups (68% of them characterized as good). Twelve out of 20 studies (60%) that reported on general characteristics of the sample, where IIV was evaluated, were characterized as good. Of note, studies reporting outliers were classified as mild according to experts’ opinion. Last, as suggested by the inverse correlation aforementioned, graphs and tables as defined in the dictionary (*Data presentation* category) were not key parameters for a comprehensive explanation of IIV. Eight out of 14 studies (57%) that presented graphs as defined in the dictionary were assessed as weak or mild. In the case of the tables, only two studies reported on additional measures of variability (min–max, interquartile range, etc.) or individual measures (responders/non-responders, producers/non-producers, etc.), and they were assessed as mild.

## Discussion

The main aim of this work was to develop a tool to support the assessment of the reporting quality of individual studies addressing either predefined or post hoc IIV in response to the consumption of plant bioactives. The developed tool, the “POSITIVe quality index” comprised 11 individual parameters classified into 4 categories and weighed/scored by criteria described in the accompanying explanatory material—the dictionary—(available at Supplementary material 2). The whole development process was performed as part of the activities of the COST Action POSITIVe and by the stepwise processes proposed by the Equator network collaborators for developers of health research reporting guidelines [27]. Other available and relevant recommendations on designing tools and general guidelines in the area of medical research, reporting on research data but also the dissemination of outcomes and medical practice [28, 29], were also taken into account.

### POSITIVe quality index parameters and recommendations

All the parameters included in the POSITIVe QI demonstrated to be useful for an adequate reporting of the IIV associated with the consumption of plant bioactives, both in bioavailability and bioactivity studies. Instead of emphasizing the parameters thoroughly described in the dictionary (Table 1), some aspects are worth discussing. During the selection of individual parameters to be included in the POSITIVe QI, individual data availability was considered a useful parameter to be reported to understand IIV fully. Moreover, as defined in the dictionary, individual data for each end-point together with the individual subject characteristics (age, gender, ethnicity, etc.) are of the greatest value, not only for understanding the IIV at a trial level but also for further data aggregation and meta-analyses [61]. Although the practice of data sharing significantly increases in the clinical research community, we found only 4 out of 30 studies on plant bioactives that provided individual data (but without additional characteristics of the subjects at the individual level) [30, 31, 52, 55]. Therefore, in order to contribute to better handling and understanding the IIV in human intervention trials, it is highly recommended for authors to prepare their data for sharing either in publications or in one of the existing research data repositories such as ClinicalTrials.gov or repositories of the Open Research Data Pilot of the European Commission, based on an adequate Data Management Plan [62]. Ohmann et al. summarized all the principles and recommendations for Individual Participant Data (IPD) sharing that should be followed in data sharing processes [63]. In case that authors decide not to share IPD, comprehensive data reporting on other categories of the QI are recommended all along the scientific process.

Regarding the study design procedure, proper *Statistics* should be taken into account, starting from the sample size calculation. An adequate calculation of the sample size is an essential element of high-quality data reporting on IIV. After the evaluation of the studies regarding power calculation and sample size, we found that conditions given in the dictionary are too demanding for this research area. There are still not enough studies, dealing with the effects of plant bioactives that reported on IIV between different groups, that authors could use to learn the population standard deviation of particular groups and related interventions. That is likely the reason why we found only one study [31] that took into consideration IIV for the sample size calculation. However, it is highly advisable to look for all studies that reported on similar results and, if they exist, to take into account reported IIV to calculate sample size.

Reporting on the distribution of data when dealing with IIV is as important as for all other data reporting cases, but we want to emphasize the importance of checking data distribution and other assumptions that should be met to get accurate statistical results. Misunderstandings of assumptions that need to be satisfied before employing parametric tests happen often. For example, the assumption for dependent *t* test that the sampling distribution of the differences between scores of two measures should be normal is usually misinterpreted by a normal distribution of scores themselves. The assumption of normal distribution within the groups, which is required when employing one-way ANOVA, is misinterpreted as the normal distribution of the total sample, etc. Assumptions for the extended list of parametric tests are explained in detail by Field et al. [64]. Visual methods for checking normality like histograms, box plots, stem-and-leaf plots, etc. could be helpful for large data sets, since statistical tests (e.g., Kolmogorov–Smirnov test, Shapiro–Wilk test, etc.) could be significant, i.e., rejecting the hypothesis of normal distribution even if deviations from normality are small. On the other hand, for small samples (< 30), statistical tests are fully necessary [65]. Shapiro–Wilk test is recommended as the best choice for testing the normality of data [66]. Then, based on data distribution, the proper statistics should be used and justified, since, for example, the presence of responders and non-responders (or producers and non-producers) may condition the data distribution. Although it could be assumed that researchers are checking the assumptions needed to use a particular test, reporting on that would indicate undeniably that those assumptions have been assessed.

Reporting on *p* value, without reporting on central tendencies and dispersion measures or the change by subgroups, is not as informative as *p* value reported together with these parameters. For example, reporting on *p* value related to different effects between men and women, as an explanation of a scatter plot, without reporting on mean and SD for each subgroup, is not as informative as it would be if authors provide these data numerically, especially if differences are small. This type of reporting cannot be used in further meta-analysis related to the IIV, unnecessarily limiting the understanding of IIV by not reporting on data that definitely exist. Moreover, these data could not be helpful for sample size calculation in future studies. Thus, reporting on *p* value should always be followed by reporting on central tendencies and dispersion measures for compared groups.

An additional parameter important for understanding the statistical significance of the effects of the intervention (*p* value) is the effect size. Even though the *p* value provides the information that effect of the intervention exists or not, it does not indicate the size of the effect (magnitude of the difference between groups/treatments) [67]. As shown in the results section, 91% of studies that reported on effect size were assessed by experts as good, regarding the quality of data reporting on IIV. Thus, it is highly recommended to report on the effect size together with the *p* value. In the studies evaluated, an informative way to report the effect size was using percent coefficient of variations, but further guidance on how to calculate and interpret an effect size for different types of analysis is summarized by Durlak et al. [68].

Although 61% of studies that presented data graphically as defined in the dictionary were assessed by experts as weak or mild, we still recommend proper graphical representation of data. As the effect size provides additional explanation to the *p* value, appropriate graphs could disclose statistics reported in tables. This is especially important for small sample sizes, as it is usually the case of nutritional intervention studies. Among the different graphs that can be used to represent IIV, scatterplots of raw data could be the most useful graphs regarding transparency of the results when dealing with small sample sizes. Such graphs could clearly show where standard deviations come from, particularly if subgroup characteristics are reported. Box plots are also a very useful way of data presentation since it shows outliers and variation.

Nevertheless, as box plot summarizes data, they are more meaningful for large sample sizes. The same applies to histograms, they are considered useful in depicting the distribution of large samples, but they are hard to understand and interpret regarding the IIV for the small samples [65, 69]. Bar charts are not recommended since they cannot say much more than a table and, moreover, they do not help reveal the distribution of data at all, since the same bar chart could be created based on different distributed data sets [69].

Another way of favorable data reporting are tables that consider criteria beyond the central tendencies and dispersion measures, including more parameters that could uncover data distribution like min–max, median, interquartile range, coefficient of variation, etc. In this way, readers get a clearer picture of data distribution and direction of variation. For instance, Brindani et al. reported on central tendencies and described the data distribution from their sample by additional parameters [70]. This type of approach may be useful to better highlight the IIV.

In the end, reporting on outliers could be considered a good way to present subjects responding in an atypical way and, thus, indicate eventual IIV. The search for homogenous data has somehow demonized outliers in research. However, when dealing with IIV, outliers can be a precious resource to better understand the differential response to the consumption of plant food bioactives and could serve to further explore the reasons behind extreme responses. Once confirmed that any potential outlier is not the result of an analytical artifact, it should be considered as robust data indicating individual variability. Their exclusion from statistical tests may be justified but reporting on them is advisable.

### Overcoming challenges in reporting inter-individual variation

The quality of reporting of clinical trials is a critical part of their overall quality as it allows readers to judge other elements in the quality domain (the design, conduction, analysis and clinical implications) and make conclusions about the reliability of their reported benefits and harms [71]. For more than two decades, an enormous effort was put by experts in clinical research into increasing the quality of reporting, with the introduction of the CONsolidated Standards Of Reporting Trials (CONSORT) Statement being a pillar. The initial statement was published in 1996 [72], further updated in 2001 [11], and 2010 [73], and supplemented with 15 official guidelines for different types of studies and 14 official extensions that address reporting of different aspects such are study designs, interventions or type of data (http://www.equator-network.org/). It has been confirmed that the introduction of these guidelines and their acknowledgment by the scientific community significantly improved the reporting quality.

There are still areas in trial reporting that remain insufficiently described and defined, with contradictory opinions or addressed even as “controversies” or (mis)uses in clinical research, such as multiplicity of data, co-variate adjustment vs. subgroup analysis, assessing individuals benefits and risks from clinical trials data, or interpretation of surprising results [24]. At the same time, most of these challenging topics are considered highly relevant for assessing the impact of IIV, using trial data for identifying those who will benefit the most, or with the least harm. For example, it is widely accepted that subgroup analyses, especially if performed post hoc, may be misleading and could bring a high risk of false conclusions that very often cannot be confirmed by subsequent studies and may have detrimental consequences [13]. However, if conducted and reported properly, analyses of IIV on trial level (e.g., effects in different subgroups) could lead to more precise recommendations, provide supporting evidence for making substantiated decisions, and could help (re)building the trust of patients/consumers [74]. Acknowledging this, the International Committee of Medical Journal Editors recommends stratifying reporting stating: “Separate reporting of data by demographic variables, such as age and sex, facilitate pooling of data for subgroups across studies and should be routine, unless there are compelling reasons not to stratify reporting, which should be explained” [75]. A list of additional sets of criteria to test the credibility of subgroup analyses includes testing whether the effect (1) is consistent across studies, (2) can it be the result of a chance, (3) is there a biological reason for the observed effect, (4) is the reported evidence from within- or between-study comparison, (5) is it defined a priory or it is a post hoc [76]. More detailed guidelines on statistical issues associated with subgroup analysis have been addressed in previous publications [73, 77] with the general conclusion that more clear and complete reporting of subgroup analyses and similar models of analyzing IIV should be encouraged. Last, efforts should also be paid in reducing, as much as possible, sources of variability not directly related to physiological conditions but to analytical constraints. In this sense, errors in the selection and measurement of some biomarkers of intake and effect that may condition the assessment of the individual response should be overcome. A deep effort and investment should be carried out in the development of standardized methodologies for the analysis of specific, reliable, and reproducible biomarkers [78, 79]. This would further favor the comparison of the reported results.

Authors’ awareness is likely another challenge to be overcome. Although the interest in the differential individual response to the consumption of plant food bioactives is growing, as demonstrated by the increasing number of publications in the field [15], the number of works dealing with this topic is rather scarce if compared to the number of publications in the field. Besides more research approaches addressing this topic from the study design, further efforts are required to report the putative IIV existing in any study. By considering the recommendations on reporting indicated in the dictionary of this QI (Table 1), authors may endow their works with valuable information on the existing IIV. Small efforts during the preparation of their manuscripts may provide a plethora of valuable information and benefits. The authors would benefit from the increased quality of their manuscripts and research, while the whole scientific community would benefit from the availability of this key information. This work presents the consensus of a significant part of the scientific community in the field. Adopting this consensus on reporting guidelines will enable the full appraisal of the trial conducted and will boost the possibilities to pool research data to gain further evidence. In summary, minor changes in the way we report data may lead to major developments in the field of plant bioactives at the individual level, moving with the times to favor suitable strategies for personalized nutrition.

Finally, although the POSITIVe QI was tailored to address the need for a better reporting of inter-individual variation in response to plant bioactives, it might be considered for non-plant bioactive compounds and as a starting point to address similar issues in other related areas, including precision medicine, public health, pharmacokinetics or toxicology [80–82]. Further validation of its use on these fields by collaborative networks is suggested.

## Electronic supplementary material

Below is the link to the electronic supplementary material.

Supplementary material 1 (XLSX 79 kb)

Supplementary material 2 (XLSX 76 kb)
